# TEP for elective primary unilateral inguinal hernia repair in men: what do we know?

**DOI:** 10.1007/s10029-019-01936-6

**Published:** 2019-05-06

**Authors:** F. Köckerling

**Affiliations:** Department of Surgery and Center for Minimally Invasive Surgery, Academic Teaching Hospital of Charité Medical School, Vivantes Hospital, Neue Bergstrasse 6, 13585 Berlin, Germany

**Keywords:** Inguinal hernia, TEP, Recurrence, Chronic pain, Postoperative complications, Costs

## Abstract

**Introduction:**

Based on the new international guidelines for groin hernia management, there is no one surgical technique that is suited to all patient characteristics and diagnostic findings. Therefore, a tailored approach should be used. Here, a distinction must be made between primary unilateral inguinal hernia in men and in women, bilateral inguinal hernia, scrotal inguinal hernia, inguinal hernia following pelvic and lower abdominal procedures, patients with severe cardiopulmonary complications, recurrent inguinal hernias and incarcerated inguinal and femoral hernias. This paper now explores the relevant studies on TEP for elective primary unilateral inguinal hernia in men, which constitutes the most common indication for repair.

**Material:**

A systematic search of the available literature was performed in February 2019 using Medline, PubMed, Scopus, Embase, Springer Link and the Cochrane Library. Only meta-analyses, systematic reviews, RCTs and comparative registry studies were considered. 117 publications were identified as relevant.

**Results:**

RCTs and comparative registry analyses demonstrated the advantages of TEP with regard to postoperative complications, complication-related reoperations, and postoperative and chronic pain compared with Lichtenstein repair for elective primary unilateral inguinal hernia repair in men. No relevant differences were found compared with TAPP. Mesh fixation is not needed in TEP, but heavyweight meshes result in a lower recurrence rate. Extraperitoneal bupivacaine analgesia vs placebo does not demonstrate any advantages, but drainage is advantageous for seroma prophylaxis. The risk of chronic pain is negatively influenced by small defects, younger patient age, preoperative pain, higher BMI, postoperative complications, higher ASA score and risk factors.

**Conclusion:**

For the subgroup of elective primary unilateral inguinal hernia in men, accounting for a proportion of less than 50% of the total collective, advantages were identified for TEP compared with open Lichtenstein repair but not versus TAPP.

## Introduction

Using evidence-based guidelines and recommendations, the international hernia societies are trying to improve the quality of hernia surgery through standardization of treatment [1–6]. The more than 100 different techniques described for repair of inguinal or femoral hernia are classified as open tissue repair, open mesh repair and laparoendoscopic mesh repair [7]. The new international guidelines of the HerniaSurge Group now only recommend the laparoendoscopic total extraperitoneal patch plasty (TEP) and transabdominal preperitoneal patch plasty (TAPP) techniques, open anterior Lichtenstein mesh repair and with limitations the mesh-free, open tissue Shouldice repair technique [6]. In that respect, the new international guidelines of the HerniaSurge Group point out that there is no one surgical technique best suited to all clinical scenarios [6].

Accordingly, the guidelines urgently recommend that surgeons adopt a tailored approach for inguinal hernia repair [6–8]. In doing so, a distinction must be made between primary unilateral inguinal hernia in men versus women, bilateral inguinal hernias, scrotal inguinal hernias, inguinal hernias after previous pelvic and lower abdominal surgery, inguinal hernias in patients with severe cardiac or pulmonary comorbidities and incarcerated inguinal hernias [6–8]. Since the proportion of women in the overall patient collective of inguinal and femoral hernias is around 10%, the proportion of recurrences is likewise around 10% and the proportion of bilateral inguinal and femoral hernias is around 20% [9, 10], elective primary unilateral inguinal hernia repair in men, accounting for a proportion of less than 50%, is the standard procedure for repair of inguinal and femoral hernias [6–8]. Due to the fact that the outcomes for repair of inguinal hernia recurrences, bilateral inguinal hernias, scrotal hernias as well as for inguinal and femoral hernias in women are less favorable [9, 11, 12], the basis used for method comparison and for performance assessment should, first of all, be an inguinal hernia repair technique based on the data available for elective primary unilateral inguinal hernia in men [6].

The data available for elective primary unilateral inguinal hernia repair in men with the TEP technique are now explored in the following.

## Materials and methods

**Fig. 1:**
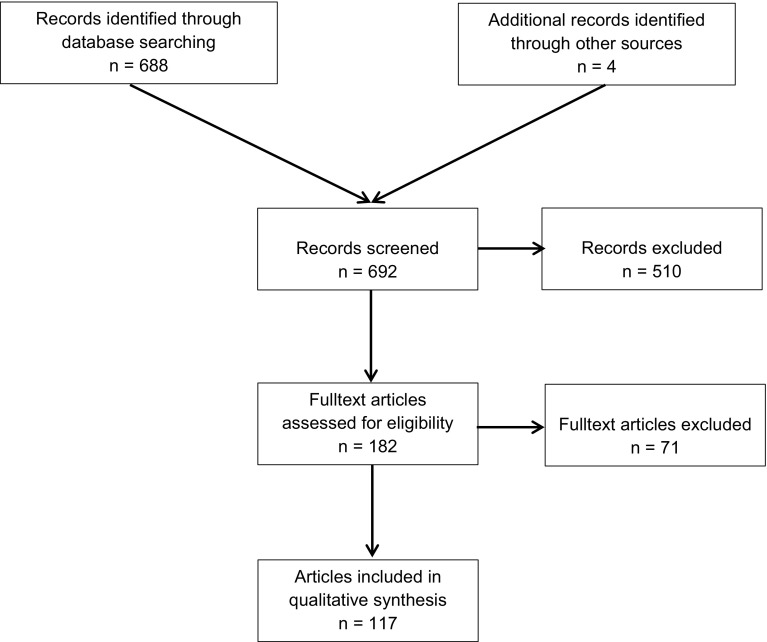
Flowchart of study inclusion

A systematic search of the available literature was performed in February 2019 using Medline, PubMed, Scopus, Embase, Springer Link and the Cochrane Library as well as a search of relevant journals and reference lists. The following search terms were used: “total extraperitoneal patchplasty”, “TEP”, “TEP hernia”, “Inguinal hernia and TEP”. The titles and abstracts of 688 publications were screened (Fig. 1).

Based on the key question, only studies reporting exclusively on elective primary unilateral inguinal hernias in men could be included. Furthermore, only studies with level of evidence 1 and 2 as per the Oxford Hierarchy of Evidence were included, i.e., meta-analyses, systematic reviews, prospective randomized controlled trials (RCTs) and comparative registry studies.

The present analysis identified 117 publications as relevant for this review. A systematic presentation and synthesis of the characteristics and findings of the included studies have been made in accordance with the Prisma guidelines [13].

## Results

### Comparison of TEP vs Lichtenstein in meta-analyses and RCTs

There are already two meta-analyses focusing exclusively on the comparison of the totally extraperitoneal patch plasty (TEP) with the Lichtenstein technique.

In a systematic review with meta-analysis and trial sequential analyses of randomized clinical trials, 5404 patients from 13 studies were included [14]. There was no significant effect of TEP compared with the Lichtenstein on the number of patients with chronic pain in a random-effects model risk ratio (RR 0.80; 95% CI 0.61–1.04; *p* = 0.09), nor was there any significant effect on the number of patients with recurrences in a random-effects model (RR 1.41; 95% CI 0.72–2.27; *p* = 0.32), and the TEP technique may or may not be associated with less severe adverse events (random-effects model RR 0.91; 95% CI 0.73–1.12; *p* = 0.37). Trial sequential analysis showed that the required information size was far from reached for important patient outcomes. The authors concluded that TEP versus Lichtenstein for inguinal hernia repair has been evaluated by 13 trials with high risk of bias. The review with meta-analyses, trial sequential analyses and error matrix approach shows no conclusive evidence of a difference between TEP and Lichtenstein on the primary outcomes chronic pain, recurrences and severe adverse events.

The meta-analysis evaluated the following RCTs: Andersson [15], Colak [16], Eklund [17], Gokalp [18], Heikkinen [19], Hildebrand [20], Merello [21], Moreno-Egea [22], Neumayer [23], Lal [24], Langeveld [25], Lau [26], and Wright [27].

Analysis of the inclusion and exclusion criteria showed for the studies [15, 16, 20–25, 27] that women or bilateral hernias or recurrent inguinal hernias were included. Hence, such studies are not suitable for comparing TEP with the Lichtenstein operation for primary unilateral inguinal hernia in male patients. As such, that leaves only the studies [17–19, 26] to answer the question addressed in this present analysis.

Another meta-analysis of randomized controlled trials comparing Lichtenstein and TEP for treatment of inguinal hernias included 13 RCTs with 3279 patients [28]. That meta-analysis also contained the studies [18, 19, 24–26] and additionally the studies of Wang [29], Kouhia [30], Eklund [31, 32], Hallen [33], Pokorny [34], Zhiping [35], Dedemadi [36] and Bringman [37].

If one compares the two meta-analyses on the basis of the included studies, one notes that only six studies [17–19, 24–26, 31, 32] were taken into account in both meta-analyses. Besides, the Eklund study [17] featured in the meta-analysis by Konig [14] takes account only of the short-term outcome, while the meta-analysis by Bobo [28] focuses only on long-term outcome [31, 32].

As pointed out above, to explore the question of comparing TEP vs Lichtenstein for primary unilateral inguinal hernia repair in men, these studies that included women, bilateral hernias and recurrent hernias had to be excluded [24, 25, 29, 30, 33–37].

Therefore, from the two meta-analyses in addition to the aforementioned studies by Eklund [17], Gokalp [18], Heikkinen [19] and Can [26], there remains only that by Eklund [31, 32].

Other studies (which were not taken into consideration in either of the two meta-analyses) which could potentially lend themselves to answering this key question included one cost analysis contained in the Eklund study [38]. In addition, there was a four-arm randomized trial comparing laparoscopic and open hernia repairs [39] as well as two studies comparing TEP under general anesthesia vs Lichtenstein under local anesthesia [40, 41]. It was not possible either to include the long-term outcome of the Langeveld study [42], since that study with 660 patients focused on bilateral inguinal hernias and recurrences.

The details and outcome of studies (1096 TEP procedures vs 1141 Lichtenstein procedures) consulted for answering the questions are listed in Table 1.

**Table 1:** Outcome of RCTs comparing TEP repair of primary unilateral inguinal hernia in men vs Lichtenstein repair

Author	Patients	Postoperative complications	Early postoperative pain	Analgesic consumption	Sick leave/return to work	Return to normal physical activity/life/domestic activity	Chronic pain	Recurrence	Cost
Eklund [17]	*n* = 665 TEP *n* = 706 Lichtenstein	The overall operative and early postoperative complication rate was 12.2% for TEP and 12.3% for Lichtenstein ns. The complication rate at 1 week was 17.3% after TEP and 17.5% after Lichtenstein ns	D1. D2. D3. D5. D7: significantly less pain for TEP; *p* < 0.001	D1. D2. D3. D5. D7: significantly less analgesic consumption for TEP; *p* < 0.001	7 days vs 12 days; *p* < 0.001	20 vs 31 days; *p* < 0.001	At 3 month: 7.6% vs 8.3%; ns	–	–
Eklund [31]	*n* = 665 TEP; *n* = 705 Lichtenstein	–	–	–			–	Median follow-up: 5.1 years (4.4-9.1) *n* = 21/600 (3.5%) TEP vs 7/583 (1.2%) Lichtenstein *p* = 0.008 Range TEP: 0-32% for surgeons and 0–13% for hospitals Range Lichtenstein: 0%-4.3% for surgeons and 0–2.4% for hospitals. Three surgeons in the TEP group were responsible for 57% of all recurrences, one of them for 33%. After exclusion of the surgeon: 1.2% vs 2.4% *p* = 0.109	–
Eklund [32]	*n* = 665 TEP *n* = 706 Lichtenstein	–	–	–	–	–	At 1 year. 11.0% vs 21.7%; *p* < 0.001. At 2 years: 11.0% vs 24.8% *p* < 0.001; At 3 years: 9.9% vs 20.2%; *p* < 0.001; At 5 years: 9.4% vs 18.8%; *p* < 0.001	–	–
Eklund [38]	*n* = 665 TEP; *n* = 705 Lichtenstein	–	–	–	–	–	–	–	Index operation: € 710.60 higher for TEP. *p* < 0.001 with recurrence and complications: €795.10 higher for TEP; *p* < 0.001 including community costs: only €292.00 higher for TEP; *p* = 0.024
Heikkinen [19]	*n* = 22 TEP; *n* = 23 Lichtenstein All employed	–	Days 1–14: Less pain for TEP *p* < 0.05	No difference in the need for analgesics: 8 vs 11 capsules; 4 vs 5 days	12 d vs 17 days; *p* = 0.01	14 d vs 20 days *p* = 0.02	–	No recurrence in either of the groups after a median follow-up of 10 months	Median hospital costs: 1.239 $ (982-1548) for TEP vs 782 $ (671–1160) for Lichtenstein *p* < 0.001 Median costs for sick leave: 2.747 $ (687-4.807) for TEP vs 3.892 $ (916-7.096) for Lichtenstein; *p* = 0.01
Dahlstrand [40]	*n* = 194 TEP; *n* = 195 Lichtenstein	–	6 weeks after surgery: Any pain in operated groin TEP 30.9% vs Lichtenstein 46.5%; *p* = 0.002	–	Sick leave exceeding 1 week: no difference	Less risk for pain affecting daily activities with TEP *p* = 0.025	–	–	–
Dhankhar [41]	*n* = 29 TEP *n* = 30 Lichtenstein	TEP *n* = 4/29 (13.8%) Lichtenstein *n* = 6/30 (20%) ns	6 h. 24 h. 48 h. 72 h. 1 week. 3 month: lower pain scores for TEP, but not significantly	Significantly more consumption of analgesics (11.3 ± 6.2 tablets of diclofenac for Lichtenstein vs 7.03 ± 5.93 tablets for TEP)	–	–	–	–	–
Lau [26]	*n* = 100 TEP *n* = 100 Lichtenstein	TEP: 15/100 (15%) Lichtenstein: 15/100 (15%) ns	D0, D1, D4, D5, D6, 0 day. 1 day. 4 days. 5 days. 6 days: pain sore at rest for the TEP group significantly lower (*p* < 0.05) Pain scores for coughing not significantly different	Postoperative analgesic requirements were comparable between TEP and Lichtenstein	8.6 days vs 14 days; *p* = 0.006	–	1 year: 9.9% for TEP vs 21.7% for Lichtenstein; *p* = 0.032	0% in TEP group and 0% in Lichtenstein group	–
Hamza [39]	*n* = 25 TEP; *n* = 25 Lichtenstein	–	Postoperative pain scores at 6 h were significantly higher in Lichtenstein repair *p* = 0.002 same at the 2nd day *p* = 0.020	–	13.22 ± 7.98 days vs. 15.25 ± 2.53 days; *p* < 0.009	7.35 ± 3.65 days vs 12.11 ± 4.23 days; *p* < 0.001	–	–	–
Gokalp [18]	*n* = 61 TEP *n* = 62 Lichtenstein	TEP *n* = 2/61 (3.3%) Lichtenstein *n* = 1/62 (1.6%) ns	VAS pain scores at 6 h, 12 h, 24 h, 48 h, 7 days, 1 month not significantly different	3.7 injections for TEP vs 4.3 injections for Lichtenstein ns	–	–	Only 1 patient in TEP group ns	Median follow-up 18 m: no recurrence in both groups	The mean total costs of the operations were significantly higher in the TEP group (975 ± 61 $) vs Lichtenstein (412 ± 34 $)

In the study by Eklund et al. [17], a total of 1513 men from 11 hospitals who presented with a primary unilateral inguinal hernia were randomized to TEP or Lichtenstein. 1371 of the 1513 men underwent surgery, 665 in the TEP group and 706 in the Lichtenstein group. The median duration of operation was 55 min for both procedures and 91.0% of the patients in both groups were discharged on the day of operation. The overall operative and postoperative complication rate was not significantly different between the two groups (TEP 12.2% vs Lichtenstein 12.3%). Patients in the TEP group experienced less postoperative pain on days 1, 2, 3 5, 7 and 14 (*p* < 0.001), consumed fewer analgesics on days 1, 2, 3, 5 and 7 (*p* < 0.001), had a shorter period of sick leave (7 versus 12 days; *p* < 0.001) and a shorter time to resumption of normal physical activity (20 versus 31 days; *p* < 0.001).

In the study by Lau et al. [26], a total of 200 male patients with primary unilateral inguinal hernia were randomized to undergo either day case unilateral TEP (*n* = 100) or open Lichtenstein (*n* = 100) hernioplasty under general anesthesia. The mean operating time for TEP (50 ± 13.2 min) was significantly shorter than for open Lichtenstein hernioplasty (58 ± 17.6 min) (*p* < 0.001). The postoperative complication rate was 15% for both procedures. The pain score at rest was significantly lower in the TEP group than in the open group on postoperative days 0,1,4,5 and 6. On average, patients returned to work 8.6 days after TEP and 14 days after Lichtenstein hernioplasty (*p* = 0.006). Postoperative recovery was comparable between the two groups.

In the study by Heikkinen et al. [19], 45 employed men with a primary unilateral inguinal hernia were randomized to undergo either a TEP (*n* = 22) or a Lichtenstein operation (*n* = 23). The operating time was shorter in the Lichtenstein group (67.5 min, range 40-88 min vs 53 min, range 42–48 min; *p* = 0.001). The mean daily pain score for 2 weeks was significantly lower for TEP (*p* < 0.05). There was no difference in the need for oral analgesics (8 vs 11 capsules) or in the duration of analgesia (4 vs 5 days). Return to normal life in the TEP group was significantly earlier (14 days vs 20 days; p = 0.02) as well as return to work (12 days vs 17 days; *p* = 0.01).

In the study by Hamza et al. [39], which is a four-arm randomized trial comparing laparoscopic (TEP, TAPP) and open (Lichtenstein, preperitoneal) hernia repairs, 50 male patients with primary inguinal hernia were randomized to TEP (*n* = 25) or Lichtenstein (*n* = 25).

The operating time for TEP, at 77.4 ± 43.21 min, was significantly longer than for Lichtenstein repair at 34.21 ± 23.5 min (*p* < 0.001). Postoperative pain on days 1 and 2 was significantly higher in patients with Lichtenstein repair (*p* = 0.002 and 0.020). TEP operations were associated with significantly faster return to normal domestic activities (7.53 ± 3.65 vs 12.11 ± 4.23; *p* < 0.001) and to work (13.22 ± 7.98 vs 15.25 ± 2.53; *p* < 0.001).

In the study by Dahlstrand et al. [40], a total of 389 men with a unilateral primary inguinal hernia were randomized to either TEP under general anesthesia (*n* = 194) or Lichtenstein under local anesthesia (*n* = 195). One patient in the TEP group and four in the Lichtenstein group were excluded due to protocol violation. Men in the TEP group had less risk of pain affecting daily activities [6/191 vs 16/187; odds ratio (OR) 0.35; 95% CI (0.13–0.91); *p* = 0.025]. Pain prevented participation in sporting activities less frequently after TEP (4.2% vs 15.5%; OR 0.24; 95% CI 0.09–0.56; *p* < 0.001). Twenty-nine patients (7.7%) reported sick leave exceeding 1 week due to groin pain, with no difference between the treatment groups. 6 weeks after surgery any pain in the operated groin was reported after TEP in 30.9% vs Lichtenstein in 46.5% (*p* = 0.002) of cases.

The authors concluded that patients who underwent the TEP procedure suffered less pain 6 weeks after inguinal hernia repair than those who underwent Lichtenstein with local anesthesia. Groin pain after Lichtenstein with local anesthesia affected the patients’ ability to perform strenuous activities such as sports more than TEP patients.

In the study by Dhankhar et al. [41], a total of 59 men with primary unilateral inguinal hernia were analyzed at the end of the study, 29 in the TEP under general anesthesia group and 30 in the Lichtenstein under local anesthesia group. The operating time (75.93 ± 13.68 vs 64.77 ± 12.66; *p* = 0.002) and total operating room time (102.66 ± 15.676 vs 72.64 ± 12.25 min; *p* < 0.001) were significantly longer in the TEP group. There was no significant difference in the postoperative complication rate (TEP 13.8% vs Lichtenstein 20%; p = ns). Postoperative pain scores in the TEP group were lower than the scores in the Lichtenstein group, but the difference was not statistically significant. There was significantly more use of analgesics and higher C-reactive protein levels in the Lichtenstein group. Quality of life and patient satisfaction were similar in both groups. The authors concluded that Lichtenstein under local anesthesia was as good as TEP under general anesthesia.

In the study by Gokalp et al. [18], 123 men with a primary unilateral inguinal hernia were treated with TEP (*n* = 61) or Lichtenstein (*n* = 62) inguinal hernia repair. The patients were followed up for a median of 18 months. In terms of postoperative pain, analgesic requirements, complications, hospital stay and duration of limitation of normal daily activities, there was no significant differences between the two groups. Operating time for TEP was 16 min longer than for Lichtenstein open tension-free technique. Return to work was shorter in patients with TEP.

Only four out of seven RCTs comparing TEP vs Lichtenstein for unilateral primary inguinal hernia in male patients reported the recurrence and chronic pain rates.

In the study by Heikkinen et al. [19], there were no recurrences in either group after a median follow-up of 10 months.

In the Lau study [26], none of the patients was found to have clinical recurrence at the 1-year follow-up assessment. The incidence of chronic pain after open repair at 1 year (21.7%) was significantly higher for Lichtenstein than for TEP (9.9%) (*p* = 0.032).

In the study by Eklund et al. [31] at a median of 5.1 (4.4–9.1) years after operation, 1275/1353 (94.2%) patients completed follow-up. The cumulative recurrence rate at 5 years was 3.5% (*n* = 21) in the TEP group and 1.2% (*n* = 7) in the Lichtenstein group (*p* = 0.008). There was wide variability in the incidence of recurrence between different surgeons and hospitals for the TEP method. The 5-year recurrence rate ranged from 0% to 32% (0/55–7/22) for the individual surgeons and from 0% to 13.5% (0/101–7/52) for the different hospitals. This was not the case for the Lichtenstein repair, where the corresponding rates ranged from 0% to 4.3% (0/46–1/23) and from 0% to 2.4% (0/64–2/86), respectively. Three out of 22 surgeons in the TEP group were responsible for 57% (12/21) of all recurrences, one of them for 33% (7/21). This surgeon operated on 25 patients, 22 of whom completed follow-up. His results diverged greatly from those of the other surgeons in the TEP group when tested for heterogeneity (*p* < 0.001). If this surgeon is excluded from the calculation, the cumulative recurrence rate in the TEP group would be 2.4%, and the difference in recurrence rate between the groups would be nonsignificant (*p* = 0.109).

The total incidence of chronic pain in the study by Eklund et al. [32] was 11.0 versus 21.7% at 1 year, 11.0 versus 24.8% at 2 years, 9.9 versus 20.2% at 3 years and 9.4 versus 18.8% at 5 years in the TEP and Lichtenstein groups, respectively (*p* < 0.001).

In the study by Gokalp et al. [18], only one case experienced persistent discomforting pain during the follow-up period. This patient in the TEP group developed genitofemoral neuralgia. In this patient, pain persisted longer than 6 months and disappeared after applying a nerve bloc three times with absolute alcohol. There has been no recurrence in either group after a median follow-up of 18 months.

Only in two studies was cost analysis performed. In the study by Gokalp et al. [18], the mean total costs of the operations were significantly higher in the TEP group (975 ± 61 US dollars) than the Lichtenstein group (412 ± 34 US dollars).

In the study by Eklund et al. [38], the total hospital costs for the index operation was € 710.6 higher for TEP repair (*p* < 0.001). Including costs associated with recurrences and complications, this difference increased to € 795.1 (*p* < 0.001). Taking community costs into account, the difference decreased by € 503.1–292.0 (*p* = 0.024).

In summary, no differences were observed in the intra- or postoperative complications following primary unilateral inguinal hernia repair in male patients between the TEP and Lichtenstein technique. Clear advantages were observed for the TEP technique in terms of early postoperative pain, analgesic consumption and return to normal daily activities and to work. When the surgeon had sufficient experience of the respective technique, i.e., after overcoming the learning curve, no significant difference was detected in the recurrence rate between the TEP and Lichtenstein operation. Likewise, chronic pain occurred significantly less often after TEP than after Lichtenstein operation. In the three RCTs with at least 100 patients in each arm, the operating time for TEP was either similar to or shorter than for Lichtenstein operation. The direct operative costs for TEP are higher than for the Lichtenstein operation. However, that difference decreases when all community costs are taken into account.

Further large RCTs are urgently needed to compare TEP versus Lichtenstein for primary unilateral inguinal hernia in male patients. It must be ensured that, by carefully selecting the participating surgeons, the learning curve has been overcome for the respective surgical technique (Table 2).

**Table 2:** Surgeons’ experience and operating time

Author	Patients	Number of participating surgeons	Experience	Operation time
Eklund [17]	*n* = 665 TEP *n* = 706 Lichtenstein	TEP: 11 hospitals, 48 surgeon 22 TEP group 26 Lichtenstein group	≥ 25 TEP No surgeon did both techniques	Median: 55 (12–180) min TEP; 55 (20–145) min Lichtenstein; ns
Lau [26]	*n* = 100 TEP; *n* = 100 Lichtenstein	–	Specialist surgeons who had experience exceeding 200 corresponding procedures	50 ± 13,2 min for TEP vs 58 ± 17,6 min for Lichtenstein; *p* < 0.001
Heikkinen [19]	*n* = 22 TEP *n* = 23 Lichtenstein All employed	1 Surgical resident	Special interesting and fair experience with open and laparoscopic hernia surgery	Median: 67,5 [72–88] min, range 40–88 min for TEP vs 53 min, range 42–78 min for Lichtenstein; *p* = 0.001
Hamza [39]	*n* = 25 TEP; *n* = 25 Lichtenstein	1 Surgeon performing all operation in a four-arm trial (TEP, TAPP, Lichtenstein, open preperitoneal	–	77.4 ± 43.21 min for TEP vs 34.21 ± 23.5 for Lichtenstein; *p* < 0.001
Dahlstrand [40]	*n* = 194 TEP; *n* = 195 Lichtenstein	2 Hospitals, 4 Surgeons	All surgeons were experienced in open and laparoscopic procedures and did not have a preference for either technique	Median 60 min, range 50–72 min for TEP, 70 min, range 60–80 min for Lichtenstein; *p* < 0.001
Dhankhar [41]	*n* = 29 TEP; *n* = 30 Lichtenstein	2 Hospitals		75.93 ± 13.68 min for TEP vs 64.77 ± 12.66 min for Lichtenstein; *p* = 0.002
Gokalp [18]	*n* = 61 TEP; *n* = 62 Lichtenstein	1 Hospital	–	62 ± 14 min for TEP vs 46 ± 11 min for Lichtenstein; *p* < 0.01

### Comparison of TEP vs Lichtenstein in registry studies

In a multivariable analysis of data from the Herniamed Registry, 10,555 Lichtenstein operations were compared with 6833 TEP operations for repair of elective primary unilateral inguinal hernia in male patients [43]. TEP was found to have advantages with regard to the postoperative complication rate (*p* < 0.001), pain at rest (*p* = 0.011), and pain on exertion (*p* < 0.001) at 1-year follow-up.

No advantages were noted for TEP in terms of the complication-related reoperation rate, recurrence rate or chronic pain rate requiring treatment at 1-year follow-up [43].

In another analysis of data from the Herniamed Registry, propensity score matching was performed to compare 12,564 TEP repairs with 12,564 Lichtenstein operations for patients with comparable characteristics [10].

That did not identify any systematic deviations between the two surgical techniques in terms of pain requiring treatment [2.8% vs 2.6%; *p* = 0.282; OR 1.090 (0.934; 1.271)] or the recurrence rate [0.8% vs 1.0%; *p* = 0.252; OR = 0.849 (0.645; 1.116)] at the 1-year follow-up [10].

However, a systematic deviation was noted with regard to the disadvantages of Lichtenstein repair in postoperative complications (3.4% vs 1.7%; *p* < 0.001), complication-related reoperation rate (1.1% vs 0.8%; *p* = 0.008) and pain at rest (5.2% vs 4.3%; *p* = 0.003) and on exertion (10.6% vs 7.7%; *p* < 0.001) [10]. On the other hand, a systematic deviation was identified with regard to the disadvantage of TEP in the intraoperative complications (0.9% vs 1.2%; *p* = 0.035).

Hence, the registry analyses demonstrated the disadvantages of TEP with regard to the intraoperative complications, but advantages for the postoperative complication rates and the complication-related reoperation rates [10]. At 1-year follow-up, TEP compared with Lichtenstein repair was found to have a lower rate of pain at rest and on exertion [10].

In summary, registry analyses identified the advantages of TEP compared with Lichtenstein operation for elective primary unilateral inguinal hernia repair in men with regard to the postoperative complications as well as complication-related reoperation and pain at rest and on exertion at the 1-year follow-up. TEP was found to have disadvantages with regard to the intraoperative complications.

### Comparison of TEP vs TAPP in meta-analyses and RCTs

There are six systematic reviews and meta-analyses available for comparison of TEP with TAPP [44–49]. The systematic reviews by McCormack [44], Wake [45] and Bracale [46] did not include enough RCTs to permit direct comparison of TEP and TAPP.

The meta-analysis by Antoniou [47] included seven RCTs by Schrenk [50], Dedemadi [36], Butler [51], Pokorny [34], Hamza [39], Gong [52] and Krishna [53] with 516 patients. However, the patient population in the Schrenk and Pokorny [34, 50] RCTs included women, the RCT by Dedemadi [36] recurrences and the RCT by Krishna [53] bilateral inguinal hernias.

The remaining RCTs by Butler [51], Hamza [39] and Gong [52] directly compared TEP and TAPP for primary unilateral inguinal hernia in men (Table 3).

**Table 3:** Outcome of RCTs comparing TEP repair of primary unilateral inguinal hernia in men vs TAPP repair

Author	Patients	Postoperative complications	Early postoperative pain	Analgesic consumption	Sick leave/return to work	Return to normal physical activity/life/domestic activity	Chronic pain	Recurrence	Cost
Butler [51]	*n* = 22 TEP *n* = 22 TAPP	–	No significant difference	No significant difference	Average number 12 days vs 12 days (ns)	–	–	4.5% for TEP and TAPP (ns)	Minimal higher ($ 125) for TEP
Hamza [39]	*n* = 25 TEP; *n* = 25 TAPP	No significant difference	Pain scores 6 h postoperative: TEP 4.8 ± 2.33 TAPP 5.8 ± 1.6 (ns)	–	TEP mean 13.2 days, TAPP mean 14.9 days (ns)	TEP mean 7.5 days, TAPP mean 9.8 days (ns)	–	4.0% for TEP and TAPP (ns)	–
Gong [52]	*n* = 52 TEP *n* = 50 TAPP	TEP 13.5% TAPP 12.0% (ns)	TEP pain score 24 h postoperative 1.7 ± 0.7 TAPP pain score 24 h postoperative 1.6 ± 0.7 (ns) TEP pain score 1 week postoperative 0.3 ± 0.5 TAPP pain score 1 week postoperative 0.3 ± 0.7 (ns)	–	–	TEP 6.6 ± 1.5 days TAPP 6.6 ± 1.7 days (ns)	–	–	No significant difference between TEP and TAPP
Günal [60]	*n* = 40 TEP *n* = 39 TAPP	TEP 7.5% TAPP 5.1% (ns)	Pain scores 6 h postoperative: TEP 5.5 ± 1.2 TAPP 6 ± 1.4 48 h postoperative: TEP 3.3 ± 1.2 TAPP 3.25 ± 1	–	–	–	–	TEP 0% TAPP 2.6% (ns)	–

Butler [51] reported minimally higher costs for TEP in comparison with TAPP. No difference was identified for postoperative pain or analgesic consumption. The average number of lost work days in both groups was 12. Likewise, there was no difference in the recurrence rate.

The RCT by Hamza [39] did not note any difference in the operating time, postoperative complications or postoperative pain between TEP and TAPP, nor was there any difference in the time to return to normal activities and work. Similarly, comparable recurrence rates were identified.

Likewise, on comparing TEP and TAPP for primary unilateral inguinal hernia in men, the RCT by Gong [52] did not find any difference in the operating time, postoperative complication rate, hospital stay or postoperative pain. The time to return to normal activities was also comparable.

The meta-analysis by Wei [48] then featured three further RCTs with a total of 1047 patients by Zhu [54], Bansal [55] and Wang [56]. The RCT by Zhu [54] investigated the effects of CO_2_ insufflation on the circulatory system and lung function and found no difference between TEP and TAPP. The RCT by Bansal [55] included a high proportion of bilateral inguinal hernias, while the surgical patient group reported on in the RCT by Wang [56] included women.

The most recent meta-analysis for comparison of TEP with TAPP by Chen [49] with 1519 randomized patients included six further RCTs by Ciftci [57], Mesci [58], Sharma [59], Günal [60], Bansal [61] and Jeelani [62]. But five of these six additional RCTs included women, recurrences or bilateral inguinal hernias [57–59, 61, 62] and therefore had to be excluded from the present analysis. The RCT by Günal [60] did not identify any clinically relevant difference between TEP and TAPP in the postoperative complications, postoperative pain or recurrence rate.

In summary, it can be stated that only very few RCTs with a small sample size are available for comparison of TEP and TAPP for elective primary unilateral inguinal hernia repair in men. Those RCTs available did not find any differences for the outcome parameters postoperative complications, postoperative pain, analgesic consumption or return to normal activities and work. More data are urgently needed for comparison of TEP and TAPP for elective primary unilateral inguinal hernia repair in men.

### Comparison of TEP and TAPP in registry studies

In a registry-based, propensity score-matched comparison of 14,426 TEP with 14,426 TAPP elective primary unilateral inguinal hernia repairs in men, no difference was seen in the intraoperative complications (1.1% vs 1.1%; *p* = 0.911), complication-related reoperation (0.9% vs 0.8%; *p* = 0.309), recurrence rate (1.0% vs 1.0%; *p* = 0.907) at 1-year follow up, pain at rest (4.8% vs 5.3%; *p* = 0.907) at 1-year follow-up, pain on exertion (8.6% vs 8.4%; *p* = 0.613) at 1-year follow-up or pain requiring treatment (2.8% vs 2.7%; *p* = 0.831) at 1-year follow-up [10]. Only for the postoperative complications (3.0% vs 1.7%; *p* < 0.001) was a significant deviation noted to the disadvantage of TAPP [10]. The higher rate of postoperative complications was due to the higher seroma rate in TAPP (2.1% vs 0.5%; *p* < 0.001). But the bleeding rate was higher in TEP at 0.8% vs 1.1% (*p* = 0.008).

In summary, a large registry analysis did not find any relevant difference between TAPP vs TEP with regard to the outcome of elective primary unilateral hernia repair in men. Only a higher seroma rate in TAPP led to a higher postoperative complication rate to the disadvantage of TAPP. Since that did not result in a higher complication-related reoperation rate, TEP and TAPP can be used with comparable safety.

### Fixation vs non-fixation of the mesh in TEP

In three meta-analyses, TEP outcomes were compared with regard to mesh fixation vs non-fixation [63–65]. All meta-analyses concluded that mesh fixation was not needed in TEP. In particular, non-fixation of the mesh was not associated with a higher recurrence rate.

The meta-analysis by Tam [63] included five RCTs by Ferzli [66], Koch [67], Moreno-Egea [68], Parschad [69] and Taylor [70] and one case-control study by Lau [71] . However, the patient collectives of all studies included women [68, 69, 71], recurrences [67, 68, 71] or bilateral inguinal hernias [66–70].

The meta-analysis by Teng [64] had only one additional study that did not report any further details of the patient collective [72].

Another RCT by Garg [73] was then included in the meta-analysis by Sajid [65], but that patient group also included bilateral inguinal hernias.

Hence, there is no RCT that compared mesh fixation vs non-fixation only for elective primary unilateral inguinal hernia repair in men.

A study based on data from the Swedish Hernia Registry identified for 1110 primary inguinal hernia repairs in men in TEP technique a low frequency of chronic pain and recurrent operations, with no difference between permanent fixation and non-permanent fixation of the mesh [74]. But that registry study, too, included a large proportion of patients with bilateral inguinal hernia. However, since the recurrence risk is higher for bilateral inguinal hernias and recurrent inguinal hernias than for primary unilateral inguinal hernia in men, the findings can be reliably extrapolated to the latter. Nonetheless, corresponding studies should also be conducted to explore that key question.

In summary, it can be stated that despite the lack of studies, it can be assumed that for primary unilateral inguinal hernia in men mesh fixation is not needed in TEP.

### Lightweight vs heavyweight mesh in TEP

Two meta-analyses are available for comparison of lightweight vs heavyweight meshes for laparoendoscopic inguinal hernia repair [75, 76]. The meta-analysis by Currie [75] included six RCTs in which the TEP technique had been used. These were RCTs carried out by Bringman [77], Heikkinen [78], Agarwal [79], Chowbey [80], Chui [81] and Peeters [82]. The authors of the meta-analysis concluded that the choice of mesh did not impact the recurrence rate or the chronic pain rate [75]. However, the RCTs reporting on the TEP technique also included patients with recurrent inguinal hernias [78] or bilateral inguinal hernias [77, 79–82]. The same studies reporting on the TEP technique were also included in another meta-analysis by Sajid [76–82]. The conclusion drawn from that meta-analysis was that on comparing lightweight vs heavyweight meshes in TEP technique, the recurrence rate did not differ but lightweight meshes resulted in a lower rate of chronic pain [76]. Based on those RCTs included in the meta-analyses, the finding cannot be applied to primary unilateral inguinal hernia in men.

Following those two meta-analyses, details of a further RCT comparing lightweight vs heavyweight meshes in 950 TEP operations for primary unilateral inguinal hernia in men were published [83]. At the 2-year follow-up a recurrence rate of 0.8% was identified for the heavyweight and of 2.7% for the lightweight meshes (*p* = 0.03) [83]. At postoperative year 1, the relevant pain rate was higher in the lightweight mesh group (2.9% vs 0.7%; *p* = 0.01) [83]. 5 years after TEP repair, the recurrence rate for the lightweight mesh continued to be significantly higher (3.8% vs 1.1%; *p* = 0.01) [84]. The authors concluded that the use of lightweight meshes in TEP did not bestow any advantages [83, 84].

The findings of that large RCT were then confirmed once again by an analysis of data from the Swedish Hernia Registry [85]. That registry analysis of data on 13,839 TEP repairs identified a significantly higher recurrence rate for lightweight meshes (4.0% vs 3.2%; *p* < 0.001) [85]. The difference persisted even after exclusion of bilateral inguinal hernias and recurrences [85].

In summary, it can be stated that the use of a heavyweight mesh for TEP repair of a primary unilateral inguinal hernia in men results in a lower recurrence rate without increasing the chronic pain rate.

### Effect of extraperitoneal bupivacaine analgesia in TEP

A meta-analysis of RCTs investigating the effect of extraperitoneal bupivacaine analgesia included eight studies with a total of 373 patients [86]. In all RCTs, TEP repair with extraperitoneal bupivacaine analgesia vs placebo was compared [87–94]. The meta-analysis did not demonstrate any advantages for extraperitoneal bupivacaine analgesia [86]. Only three of the eight included RCTs investigated the effect of bupivacaine in primary unilateral inguinal hernia repair in men [92–94]. Likewise, these three RCTs did not identify any advantage for administration of extraperitoneal analgesia on concluding TEP repair.

In summary, it can thus be noted that extraperitoneal bupivacaine analgesia does not have any advantages in TEP.

### Drainage after TEP

In one RCT with 90 patients, TEP repair of primary unilateral inguinal hernia with drainage vs non-drainage was compared [95]. Drainage was found to be associated with a significant reduction in the seroma rate up to postoperative day 6 [95]. The authors concluded that drainage of the extraperitoneal space in TEP reduced the seroma rate in the early postoperative phase [95].

### Convalescence after TEP

A systematic review then demonstrated that the risk factors fixation vs non-fixation, heavyweight vs lightweight mesh and peritoneal bupivacaine analgesia vs saline had no effect on the convalescence of patients after primary unilateral inguinal hernia repair in men with the TEP technique [96].

### Influencing factors for chronic pain in TEP

A systematic review of early pain after laparoendoscopic inguinal hernia repair found that TEP was associated with the greatest pain intensity on postoperative day 1 [97], with the greatest pain intensity observed in young men [97]. The rate of moderate to severe chronic pain identified in a systematic review after laparoendoscopic repair was 1.1% [98].

An analysis of data for 57,999 male patients from the Herniamed Registry who underwent elective primary unilateral inguinal hernia repair revealed that small inguinal hernia, independently of the surgical technique, was associated with a significantly higher risk of chronic pain requiring treatment [99]. Comparison of EHS I (< 1.5 cm) vs EHS II (≥ 1.5–3 cm) [OR 1.482 (1.212–1.812); *p* < 0.001] and EHS I (< 1.5 cm) vs EHS III (> 3 cm) [OR 1.582 (1.199–2.088); *p* = 0.001] in TEP demonstrated that small hernia presented a significantly higher risk for development of chronic pain requiring treatment [99].

Similarly, a higher probability of chronic inguinal pain requiring treatment in relation to patient age (< 55 years vs ≥ 55 years) was identified once again in the registry analysis [OR 2.021 (1.806–2.201); *p* < 0.001] [99]. Other negative influencing factors were preoperative pain, higher BMI, postoperative complications, higher ASA score and risk factors [99].

### Male infertility following TEP

One systematic review investigated the influence of TEP on male infertility [100]. The analysis included 108 TEP repairs reported on in the studies by Skawran [101] and Peeters [102]. In both studies bilateral inguinal hernias were repaired with the TEP technique. Likewise, in the study protocol by Schouten on male infertility after TEP inguinal hernia repair, only bilateral inguinal hernias were included [103].

Likewise, another systematic review by Dong [104] featured the studies by Skawran [101], Peeters [102] and the study protocol by Schouten, in addition to the studies by Lal [105], Singh [106], Akbulut [107] and Peeters [82]. But these additional studies, too, included bilateral inguinal hernias.

From that systematic review, the authors concluded that inguinal hernia repair with mesh in laparoendoscopic technique had no significant effect on male fertility [104]. Although all the included studies featured bilateral inguinal hernias, it can be assumed that the conclusion drawn can also be applied to elective primary unilateral inguinal hernia repair in male patients, since the extent of dissection is less in primary unilateral inguinal hernia than in bilateral repair.

### Surgeon volume in the outcome of TEP

Systematic reviews have demonstrated strong evidence of an association between higher volumes and better outcome in surgery [108].

A study of data from the Herniamed Registry identified for primary unilateral inguinal hernia repair in men in laparoendoscopic technique significant differences in relation to the annual surgeon volume [109]. Multivariable analysis revealed that patients operated on by surgeons with an annual surgeon volume of ≥ 25 operations had a significantly lower risk of recurrence [< 25 vs ≥ 25: OR 1.494 (1.056–2.115); *p* = 0.023] and pain on exertion [< 25 vs ≥ 25: OR 1.191 (1.062–1.337); *p* = 0.003] at the 1-year follow-up [109].

That finding was confirmed by a further study for surgeons with > 30 TEP operations per year, albeit that study included a very large proportion of bilateral procedures [110].

Likewise, the study by Aikoye [111], which also included bilateral inguinal hernias, confirmed the relationship between surgical volume and outcome in TEP inguinal hernia repair.

### Personal experience with the TEP

As the chairman responsible for a Department of General Surgery, first in Hanover and then in Berlin, the author has 20 years’ experience of routine inguinal hernia repair in TEP technique [112]. During that period, the technique was standardized in accordance with evidence-based data [3, 4, 112–114]. The findings from the time in Hanover have been reported in several publications [115–117]. In a consecutive series of 5203 TEP repairs in 3868 patients with inguinal hernias (uni- and bilateral in men and women, recurrences), the intraoperative complication rate was 0.9%, the postoperative complication rate 3.4%, the complication-related reoperation rate 2.8% and the recurrence rate 0.6% [113].

Between 2010 and 2018, 3365 hernia patients were treated in the Certified Hernia Center, Department of General Surgery, Vivantes Hospital Berlin, and their data entered into the Herniamed Hernia Registry. These related to 1679 patients with 2166 inguinal hernia repairs, 761 incisional hernias, 375 epigastric hernias, 283 umbilical hernias, 239 hiatal hernias and 28 parastomal hernias. Of the 2166 inguinal hernia repairs, 1000 were performed or assisted with TAPP technique by two senior physicians and 834 with TEP technique, which were all carried out by the author himself or at which he assisted, 291 with Lichtenstein and 41 with other techniques.

Of the 834 TEP repairs, only 196 (23.5%) involved elective primary unilateral inguinal hernia repair in men. No intraoperative complications occurred in that subgroup of male patients with unilateral inguinal hernia. In the postoperative phase, there were three cases (1.5%) of secondary bleeding in patients continuing to receive treatment with platelet aggregation inhibitors, two cases of seroma (1.0%) and two (1.0%) of impaired wound healing at a trocar puncture site. The complication-related reoperation rate was 1.0%. This was because of secondary bleeding. At the 1-year follow-up, no patient suffered from chronic pain requiring treatment and there were no recurrences. Pain at rest was reported by 2.0% of patients and pain on exertion by 7.7%. Hence, through standardization of the TEP technique, it is possible to achieve very good perioperative outcomes and low chronic pain and recurrence rates. As the same is proven for the TAPP technique, laparoendoscopic repair is the standard procedure for elective primary unilateral inguinal hernia in men in our hospital.

## Discussion

In all guidelines TEP and TAPP as well as the Lichtenstein operation as a mesh procedure are recommended for repair of inguinal hernia [1–6]. However, the new international guidelines for groin hernia management state that there is no one technique that is suited to all inguinal hernia findings [6]. Rather, it is recommended that a tailored approach should be used based on the surgeon’s expertise, the local/national resources and on patient- and hernia-related factors. Accordingly, in line with the tailored approach concept based on patient- and hernia-related factors, a distinction must be made between primary unilateral inguinal hernia in men and in women, primary bilateral inguinal hernia in men and in women, primary scrotal hernia, inguinal hernia after pelvic and lower abdominal procedures, inguinal hernia in patients with severe cardiopulmonary risk factors, recurrent inguinal hernias and incarcerated inguinal hernias [6–8]. These subgroups from the entire collective of inguinal hernias should in the future be scientifically viewed as separate entities. The reason for this is that there are significant differences in the outcomes of inguinal hernia surgery between the subgroups [6, 9, 11, 12]. Elective primary unilateral inguinal hernia in men accounting for about 50% of inguinal hernias is the largest subgroup, which explains why their repair constitutes the standard procedure in inguinal hernia surgery [6–8, 10]. The proportion of primary unilateral inguinal hernias in women is around 10%, recurrent hernias likewise account for 10% and bilateral inguinal hernias for around 20% [6–12].

A rigorous scientific reduction to subgroups from the entire collective of inguinal hernias not only results in exclusion of several RCTs, but also in a re-evaluation of systematic reviews and meta-analyses. This considerably reduces the total number of studies available for answering key scientific questions. But this would mean that the remaining studies would enable more precise statements to be issued for a specific subgroup of inguinal hernias.

In the present analysis of the outcome of elective primary unilateral inguinal hernia repair in men using TEP technique, ten publications [17–19, 26, 31, 32, 38–41] from seven RCTs demonstrated advantages for TEP in comparison with open Lichtenstein repair. Clear advantages have been observed for the TEP technique in terms of early postoperative pain, analgesic consumption and return to normal daily activities and to work. Likewise, chronic pain occurred significantly less often after TEP than after Lichtenstein repair. No difference was found in the postoperative complications or recurrence rates.

Unlike in the RCTs, registry analyses identified for Lichtenstein repair a significantly higher postoperative complication rate and complication-related reoperation rate in comparison with TEP. This could also be because of no patient selection in the registries compared with the rigorous patient selection in the RCTs. Risk patients are not excluded from registries. Similarly, selection of the participating surgeons is less strict in registries than in RCTs. As regards chronic pain, advantages were identified for TEP compared with Lichtenstein repair in the available RCTs and registry analyses. An overview of the available findings for TEP vs Lichtenstein for elective primary unilateral inguinal hernia in men demonstrated advantages for TEP with regard to postoperative complications, complication-related reoperations, early postoperative pain, return to normal activity and work as well as chronic pain. No difference was found in the recurrence rate. A higher intraoperative complication rate may be expected with TEP.

Comparison of TEP vs TAPP did not find any relevant difference [10, 39, 51, 52] in either the RCTs or registry data for TEP vs TAPP in primary unilateral inguinal hernia repair in men. Only in one registry analysis [10] was a higher seroma rate identified for TAPP, leading to a higher postoperative complication rate but without increasing the complication-related reoperation rate. This is thought to have been attributable to the failure to reduce the medial hernia defect [6].

There are no RCTs or registry analyses available for mesh fixation vs non-fixation in TEP for elective primary unilateral inguinal hernia repair in men. But from the findings available for bilateral inguinal hernias it can be concluded that fixation can be dispensed with in elective primary unilateral inguinal hernia repair in men [63–74].

Surprisingly, comparison of lightweight vs heavyweight meshes for elective primary unilateral inguinal hernia repair in men demonstrated an advantage for the heavyweight meshes in terms of a lower recurrence rate [75–85], with no attendant increase in the chronic pain rate.

Extraperitoneal bupivacaine analgesia vs placebo did not identify any positive effect following elective primary unilateral inguinal hernia TEP repair and should therefore not be administered [86–94].

None of the risk factors, fixation vs non-fixation, lightweight vs heavyweight mesh or preperitoneal bupivacaine analgesia impacted convalescence after elective primary unilateral inguinal hernia repair in men using TEP technique [96].

For smaller defects, an increased risk of chronic inguinal pain was identified, independently of the surgical technique, following elective primary unilateral inguinal hernia repair in men [99]. Other influencing factors were age < 55, preoperative pain, higher BMI, postoperative complications, high ASA score and risk factors [99].

While there are no studies on male infertility following elective primary unilateral inguinal hernia TEP repair in men, as these are available only for patients operated on for bilateral hernias, the findings for bilateral TEP can be extrapolated to unilateral repair since this involves less dissection [82, 101–107].

An annual surgeon volume von ≥ 25 TEP operations for elective primary unilateral inguinal hernia repair in men results in a significant reduction in the risk of recurrence and pain on exertion [109].

In summary, it can be stated that in the future scientific studies aimed at comparison of different surgical techniques and identification of factors influencing the outcome should focus on, as far as possible, homogeneous subgroups of inguinal hernias. The subgroup of elective primary unilateral inguinal hernia in men is best suited to that purpose, since it is the most common type of inguinal hernia, accounting for a proportion of around 50% of the entire collective of inguinal hernias and constituting the standard procedure in inguinal hernia surgery. The present analysis of TEP for this subgroup demonstrates advantages compared with open Lichtenstein repair and comparable findings with the TAPP. Mesh fixation is not needed in TEP, but heavyweight meshes result in a lower recurrence rate. Extraperitoneal bupivacaine analgesia does not demonstrate any advantages for postoperative pain, but drainage appears to reduce the seroma rate. Mesh non-fixation, the use of a heavyweight mesh or preperitoneal bupivacaine analgesia do not have a positive effect on convalescence. The risk of chronic pain following TEP is increased for smaller defects, younger patients, preoperative pain, higher BMI, postoperative complications, higher ASA score and risk factors. TEP was not found to have a negative effect on male infertility. An annual surgeon volume of ≥ 25 TEP repairs results in lower recurrence and pain on exertion rates.
